# Dropless cataract surgery: comparing sub-Tenon’s and topical steroids for postoperative inflammation prophylaxis

**DOI:** 10.1038/s41433-026-04309-3

**Published:** 2026-02-19

**Authors:** Alan Y. Huang, Nitya Rao, Michael E. Sulewski, Stephen Armenti

**Affiliations:** 1 Perelman School of Medicine, University of Pennsylvania, Philadelphia, PA USA; 2 Department of Ophthalmology, Atrium Wake Forest Baptist, Winston, Salem, NC USA; 3 Department of Ophthalmology, Scheie Eye Institute, University of Pennsylvania, Philadelphia, PA USA

**Keywords:** Outcomes research, Surgery

## Abstract

**Background/objectives:**

To compare sub-Tenon’s (“dropless”) and topical steroid regimens for inflammation prophylaxis following cataract surgery.

**Subjects/methods:**

This retrospective cohort study was conducted using the electronic health record (EHR) of a single academic medical centre in Philadelphia, Pennsylvania, USA. All patients who underwent phacoemulsification cataract surgeries from January 1, 2023, to August 31, 2024 were analysed. Eyes in the dropless group received an intraoperative sub-Tenon’s triamcinolone acetonide (TA) injection. Eyes in the topical group followed a routine postoperative topical steroid taper (prednisolone acetate 1% drops). Baseline characteristics included age, race, and relevant comorbidities. Key outcomes assessed were incidence of intraocular pressure (IOP) spikes greater than 35 mmHg, cystoid macular oedema (CMO), and rebound iritis. Differences were analysed with Fisher’s exact tests and linear regressions.

**Results:**

A total of 3307 eyes were included (291 treated with the dropless protocol and 3016 with topical drops). Baseline patient characteristics were similar between groups in terms of age, sex, and comorbidities, with the exception that the dropless cohort had a higher proportion of Black patients (46.0% vs 34.4%, *p* < 0.01) and diabetic patients (40.5% vs. 30.9%, *p* < 0.01). There were no significant differences between dropless and topical groups in the incidence of IOP spikes >35 mmHg, CMO, or rebound iritis (all *p* > 0.05).

**Conclusions:**

Sub-Tenon’s TA was noninferior to topical steroids in terms of incidence of IOP spikes, CMO, and rebound iritis following cataract surgery. However, further research is needed to identify risk factors for adverse outcomes and determine ideal use cases for a dropless protocol.

## Introduction

Over 28 million cataract surgeries are performed each year, making it one of the most common surgeries performed worldwide [1]. Standard postoperative care after cataract surgery includes several weeks of topical eye drops, typically including antibiotics and steroids for infection and inflammation prophylaxis, respectively [2]. These medication regimens also often require patients to taper certain drops over varying periods of time. Given these complexities, patients often face barriers with medication adherence, proper drop technique, and dosing errors [3, 4]. In recent years, there has been growing interest in “dropless” cataract surgery techniques in which antibiotics or steroids are injected intraoperatively as a depot or via drug-eluting implants, thus minimising the need for postoperative drops and simplifying patient instructions [5]. This approach could not only improve patient outcomes but also decrease overall health system costs [6].

Current evidence strongly supports the use of intracameral antibiotics at the time of surgery, which has been shown to significantly reduce the risk of postoperative endophthalmitis without need for additional topical antibiotics [2, 7]. However, the evidence for intraocular steroids to control postoperative inflammation has been less conclusive, as the risk of intraocular pressure (IOP) spikes remains a concern with steroid use [2, 3, 8]. Recent studies have explored various intraocular locations for steroid injection (e.g., anterior chamber vs. intravitreally), as well as sustained-release formulations and inserts [9–12]. However, further research is needed to understand the risks and benefits of a dropless approach to cataract surgery across a wide variety of patient populations and care settings.

This study examines outcomes of patients undergoing cataract surgery at a single academic medical centre in a large city, comparing patients who received sub-Tenon’s triamcinolone acetonide (TA) as the sole postoperative inflammation prophylaxis and those who received traditional steroid drop regimens. Assessing differences between postoperative outcomes for cataract surgery can help inform clinical practice and improve care for the millions of patients undergoing this procedure worldwide.

## Methods

### Study design

This retrospective cohort analysis assessed phacoemulsification cataract surgeries performed at the Scheie Eye Institute, the ophthalmology department of University of Pennsylvania Health Systems (Philadelphia, PA). The study period spanned from January 1, 2023, through August 31, 2024. All cataract surgeries during this interval were identified via the electronic health record (EHR) system using procedural and diagnosis codes. The study adhered to the tenets of the Declaration of Helsinki and was approved by the University of Pennsylvania Institutional Review Board (IRB) (protocol number 856312).

### Study population

Inclusion criteria for this study were phacoemulsification cataract surgeries performed during the study period. Only patients age 18 or older were included. Only the first operative eye for each patient was assessed. Cases were excluded if they had any concurrent procedures (e.g., cataract surgery with trabeculectomy). Patients were excluded from the analysis if they had a documented history of macular oedema or iritis.

### Primary exposure

Medication records from the EHR were assessed to determine dropless and topical treatment groups. The dropless group consisted of patients who received intraoperative sub-Tenon’s TA during cataract surgery, without exposure to topical steroids postoperatively. At this centre, the standard dose was 4 to 10 mg (0.5 mL of 10–20 mg/mL) of TA injected into the sub-Tenon’s space. The topical treatment group was defined as those who received topical prednisolone acetate, most commonly delivered via 1% drops or a combined Prednisolone-Moxifloxacin-Bromfenac 1-0.5-0.075% combination solution. Tapering regimens typically required patients to administer drops four times daily for the first week and then gradually taper over 3–4 weeks. Topical nonsteroidal anti-inflammatory drugs (NSAIDS) were given to patients on a case by case basis, most often in the form of ketorolac tromethamine 0.5% solution.

### Patient characteristics

Baseline characteristics of patients assessed included age, gender, race, body mass index (BMI) at time of surgery, and best corrected visual acuity (BCVA). BCVA of the surgical eye was assessed using the most recent measurement prior to the procedure and the first measurement at least one month after the procedure to account for vision fluctuations immediately following surgery. BCVA was converted from Snellen to log of the minimum angle of resolution (logMAR) ignoring letters gained or missed (e.g., +1 or −1). Additional visual acuity measurements were converted using the following conversions: counting fingers (CF) = logMAR 1.9, hand motion (HM) = logMAR 2.3, light perception (LP) = logMAR 2.7, and no light perception (NLP) = logMAR 4 [13].

Existing medical conditions assessed for each patient included diabetes, glaucoma, presence of an epiretinal membrane, and diabetic retinopathy. Diagnoses were determined using International Classification of Diseases, Tenth Revision (ICD-10) codes. The date of diagnosis was used to determine preexisting conditions versus diagnoses made after the procedure. All types of diabetes were included. Glaucoma diagnoses excluded neovascular glaucoma, phacomorphic glaucoma, and traumatic glaucoma.

### Outcomes

Key outcomes assessed included IOP spikes >35 mmHg in the surgical eye at any postoperative visit between 4 days to 3 months after the surgery, cystoid macular oedema (CMO), and rebound iritis. For IOP spikes, IOP measurements from postoperative days 0–3 were excluded, as IOP spikes in the immediate postoperative period are most often related to surgical disruption and not steroid exposure. CMO was first determined using text matching for keywords within postoperative appointment notes and then manually confirmed using notes from optical coherence tomography (OCT) results. Rebound iritis was similarly determined using text matching for keywords within postoperative appointment notes and then manually confirmed by reviewing appointment notes. Additionally, secondary analyses were completed to assess differences in adverse outcomes (IOP spike >35 mmHg, CMO, and rebound iritis) between patients with and without prior diagnoses of glaucoma and diabetes.

### Statistical analyses

All statistical analyses were performed using R version 4.5.0 and Stata/SE 18 (College Station, TX). Differences between dropless and topical treatment groups were assessed using linear regressions for continuous variables and Fisher’s exact test for categorical variables. *P*-values less than 0.05 (two-tailed) were considered statistically significant.

## Results

### Baseline patient characteristics

Baseline demographics and characteristics of our study population are outlined in Table 1. A total of 3307 cataract surgeries met the inclusion criteria, of which 291 (8.8%) were managed with the dropless protocol and 3016 (91.2%) received the standard topical drop regimen. The dropless group had a statistically lower mean age of 68.7 years compared with the mean age of 71.3 years in the topical group (*p* < 0.01). Both groups had more female patients than male (62%, *p* = 1.00). The racial composition differed between groups: the dropless group had a significantly higher percentage of Black patients (46.0%) compared to the topical group (34.4%) and lower percentage of White patients (35.1% vs. 52.3%) (*p* < 0.01). BMI was similar between dropless and topical groups (*p* = 0.92). The prevalence of preexisting glaucoma and epiretinal membrane was similar between dropless and topical groups (*p* = 0.22 and 0.21, respectively). The dropless group had a higher prevalence of preexisting diabetes (40.5% vs. 30.9%, *p* < 0.01) and diabetic retinopathy (13.7% vs. 9.4%, *p* = 0.02). BCVA was worse in the dropless group both before surgery (logMAR 0.580 vs. 0.414, *p* < 0.01) and after surgery (logMAR 0.215 vs. 0.172, *p* = 0.04). However, the calculated improvement in BCVA after surgery from baseline was greater in the dropless group (change in logMAR of 0.340 vs. 0.241, *p* < 0.01).

**Table 1:** Baseline characteristics of patients who received cataract surgery from January 1st, 2023 – August 31st, 2024.

	Topical drugs	Sub-Tenon’s TA	Total	Test^a^
*N*	3016 (91.2%)	291 (8.8%)	3307 (100.0%)	
Age	71.250 (9.826)	68.670 (10.243)	71.023 (9.889)	<0.01
Gender				
Female	1861 (61.7%)	180 (61.9%)	2041 (61.7%)	1.00
Male	1155 (38.3%)	111 (38.1%)	1266 (38.3%)	
Race				
Black	1036 (34.4%)	134 (46.0%)	1170 (35.4%)	<0.01
White	1576 (52.3%)	102 (35.1%)	1678 (50.7%)	
Other	404 (13.4%)	55 (18.9%)	459 (13.9%)	
BMI	29.827 (87.876)	29.300 (7.423)	29.780 (83.910)	0.92
Diabetes	932 (30.9%)	118 (40.5%)	1050 (31.8%)	<0.01
Glaucoma	132 (4.4%)	8 (2.7%)	140 (4.2%)	0.22
Epiretinal membrane	160 (5.3%)	10 (3.4%)	170 (5.1%)	0.21
Diabetic retinopathy	285 (9.4%)	40 (13.7%)	325 (9.8%)	0.02
BCVA of surgical eye before surgery	0.414 (0.455)	0.580 (0.600)	0.427 (0.470)	<0.01
BCVA of surgical eye after surgery	0.172 (0.313)	0.215 (0.426)	0.177 (0.327)	0.04
Change in BCVA of surgical eye	0.241 (0.427)	0.340 (0.572)	0.249 (0.441)	<0.01

*TA* triamcinolone acetonide, *BMI* body mass index, *BCVA* best corrected visual acuity.

^a^Linear regression used for comparisons of continuous variables and Fisher’s exact test for comparisons of categorical variables.

### Postoperative outcomes

Table 2 summarises differences in postoperative adverse outcomes in each group. There were no statistically significant differences in postoperative IOP spikes, CMO, or rebound iritis between groups (*p* = 0.188, 0.05, and 0.16, respectively).

**Table 2:** Adverse outcomes of patients who received cataract surgery from January 1st, 2023 – August 31st, 2024.

	Topical drugs	Sub-Tenon’s TA	Total	Test^a^
*N*	3016 (91.2%)	291 (8.8%)	3307 (100.0%)	
IOP spikes > 35 mmHg				
No	2990 (99.1%)	286 (98.3%)	3276 (99.1%)	0.188
Yes	26 (0.9%)	5 (1.7%)	31 (0.9%)	
Cystoid macular oedema				
No	2926 (97.0%)	276 (94.8%)	3202 (96.8%)	0.05
Yes	90 (3.0%)	15 (5.2%)	105 (3.2%)	
Rebound iritis				
No	2900 (96.2%)	275 (94.5%)	3175 (96.0%)	0.16
Yes	116 (3.8%)	16 (5.5%)	132 (4.0%)	

*TA* triamcinolone acetonide.

^a^Fisher’s exact test used for comparisons of categorical variables.

### Outcomes in patients with glaucoma

Results from secondary analyses of patients with and without preexisting glaucoma are outlined in Table 3. Fewer patients with glaucoma also had a history of diabetes (18.6% vs. 32.3%, *p* < 0.01). There were no significant differences in patients with glaucoma with regard to race, postoperative treatment protocol, and adverse outcomes (IOP spike >35 mmHg, CMO, and rebound iritis) (all *p* > 0.05).

**Table 3:** Factors and outcomes associated with glaucoma in study population.

	No history of glaucoma	Existing glaucoma diagnosis	Total	Test^a^
*N*	3167 (95.8%)	140 (4.2%)	3307 (100.0%)	
Diabetes				
No	2143 (67.7%)	114 (81.4%)	2257 (68.2%)	<0.01
Yes	1024 (32.3%)	26 (18.6%)	1050 (31.8%)	
Race				
Black	1114 (35.2%)	56 (40.0%)	1170 (35.4%)	0.21
White	1607 (50.7%)	71 (50.7%)	1678 (50.7%)	
Other	446 (14.1%)	13 (9.3%)	459 (13.9%)	
Sub-Tenon’s TA				
No	2884 (91.1%)	132 (94.3%)	3016 (91.2%)	0.22
Yes	283 (8.9%)	8 (5.7%)	291 (8.8%)	
IOP spikes > 35 mmHg				
No	3138 (99.1%)	138 (98.6%)	3276 (99.1%)	0.380
Yes	29 (0.9%)	2 (1.4%)	31 (0.9%)	
Cystoid macular oedema				
No	3065 (96.8%)	137 (97.9%)	3202 (96.8%)	0.63
Yes	102 (3.2%)	3 (2.1%)	105 (3.2%)	
Rebound iritis				
No	3039 (96.0%)	136 (97.1%)	3175 (96.0%)	0.66
Yes	128 (4.0%)	4 (2.9%)	132 (4.0%)	

*TA* triamcinolone acetonide

^a^Fisher’s exact test used for comparisons of categorical variables.

### Outcomes in patients with diabetes

Results from secondary analyses of patients with and without preexisting diabetes are outlined in Table 4. A higher proportion of Black patients had existing diabetes diagnoses at time of surgery (58.0% vs. 24.9%, *p* < 0.01). More patients with diabetes received the dropless postoperative treatment protocol (11.2% vs. 7.7%, *p* < 0.01). There were no differences in IOP spikes (*p* = 0.439), but patients with diabetes had higher rates of CMO (6.7% vs. 1.6%) and rebound iritis (5.7% vs. 3.2%) (all *p* < 0.01).

**Table 4:** Factors and outcomes associated with diabetes in study population.

	No history of diabetes	Existing diabetes diagnosis	Total	Test^a^
*N*	2257 (68.2%)	1050 (31.8%)	3307 (100.0%)	
Race				
Black	561 (24.9%)	609 (58.0%)	1170 (35.4%)	<0.01
White	311 (13.8%)	148 (14.1%)	459 (13.9%)	
Other	1385 (61.4%)	293 (27.9%)	1678 (50.7%)	
Sub-Tenon’s TA				
No	2084 (92.3%)	932 (88.8%)	3016 (91.2%)	<0.01
Yes	173 (7.7%)	118 (11.2%)	291 (8.8%)	
IOP spikes > 35 mmHg				
No	2238 (99.2%)	1038 (98.9%)	3276 (99.1%)	0.439
Yes	19 (0.8%)	12 (1.1%)	31 (0.9%)	
Cystoid macular oedema				
No	2222 (98.4%)	980 (93.3%)	3202 (96.8%)	<0.01
Yes	35 (1.6%)	70 (6.7%)	105 (3.2%)	
Rebound iritis				
No	2185 (96.8%)	990 (94.3%)	3175 (96.0%)	<0.01
Yes	72 (3.2%)	60 (5.7%)	132 (4.0%)	

*TA* triamcinolone acetonide.

^a^Fisher’s exact test used for comparisons of categorical variables.

## Discussion

In this retrospective cohort study, we assessed differences in baseline characteristics and adverse outcomes of a dropless cataract surgery protocol—in which postoperative inflammation prophylaxis consisted of sub-Tenon’s TA only—compared to a traditional topical steroid regimen. We found that the dropless cohort had no differences in incidence of postoperative IOP spikes, CMO, or rebound iritis. These findings indicate that sub-Tenon’s TA was effective at postoperative inflammation prophylaxis and was not associated with increased rates of IOP spikes. Understanding these risks and benefits can help inform future clinical practice guidelines when determining the ideal approach to postoperative care for cataract surgery.

The dropless approach proved noninferior to the traditional topical approach for controlling postoperative inflammation given no significant differences in incidence of CMO and rebound iritis between groups. This suggests that the single sub-Tenon’s TA depot provided sufficient anti-inflammatory coverage through the postoperative period, comparable to a tapered course of topical steroids. Prior studies have similarly reported that intraoperative steroid injections can match or even provide greater postoperative inflammation prophylaxis than topical steroids [14–16]. For CMO, the numeric difference we observed (5.2% vs 3.0%, *p* = 0.05) could suggest a trend toward a slight increase in CMO incidence with sub-Tenon’s TA, but this did not reach statistical significance. A contributing factor for this finding may be the higher rates of diabetes and diabetic retinopathy in our dropless cohort, both of which are associated with increased risk of CMO. There were also higher rates of CMO among diabetic patients across the entire study population. However, our CMO incidence for both groups are within the expected ranges for post-cataract surgery CMO of 0.1–12% [17, 18]. Our cohort had higher rates of rebound iritis compared to previous studies, which have reported 1–2% incidence of rebound iritis after cataract surgery [15, 19]. This may be explained by the higher proportion of Black patients in our study cohort, as previous studies have demonstrated higher risk of rebound iritis in Black patients [19, 20]. We also observed higher rates of rebound iritis among diabetic patients, which represented over half of the Black patients in this study; future studies should include larger sample sizes to perform multivariate analysis to determine which factors are driving this association. Taking these findings together, our study further demonstrates the noninferiority of dropless cataract surgery in inflammation prophylaxis in a more diverse patient population. This is important given documented differences in postoperative outcomes of cataract surgery between racial and socioeconomic groups, likely a result of complex social determinants of health [21–23].

One concern with intraoperative steroid use is steroid-related IOP spikes [2, 3]. We found no significant differences in the incidence of IOP spikes >35 mmHg in the dropless group compared to the topical group. Intraocular steroids are used with caution because a depot injection delivers a higher total steroid load to the eye that cannot be tapered or removed, which in susceptible individuals can precipitate steroid-related IOP spikes [24–26]. Topical steroids, by contrast, can be tapered or stopped at the first sign of IOP increase, potentially mitigating IOP spikes. Our observation is corroborated by a retrospective study of nearly seventy thousand patients seen at Kaiser Permanente Northern California, which found that patients injected with subconjunctival TA as the sole inflammation prophylaxis had a similar risk of IOP spikes as topical treatment (defined as IOP > 30 mmHg or 10 mmHg above baseline) [15]. However, Wu et al. reported a higher steroid response rate in dropless patients in a study of 368 eyes (defined as IOP > 24 mmHg or 50% above baseline) [27]. These discrepancies may be explained by differences in sample size and by the higher IOP spike cutoffs used in our study (>35 mmHg) and the Kaiser Permanente study (>30 mmHg). Notably, there were no differences in preexisting glaucoma diagnoses between the dropless and topical groups in our study, further supporting the safety of sub-Tenon’s TA even in patients with glaucoma. Current literature suggests that there are no concrete ways to determine steroid response prior to surgery [26, 28, 29]. Thus, close monitoring of postoperative IOP should still be prioritised in all patients who receive periocular steroids during cataract surgery until identifiable risk factors are determined.

The results of this study have direct clinical implications. First, they show that in a real-world setting, a dropless regimen could decrease the need for postoperative drops without additional inflammation-related complications such as CMO and iritis. This is especially relevant for populations with barriers to medication adherence (e.g., patients with housing instability or those with fine motor skill difficulty) [30–32]. Previous studies have shown that patients have a high rate of incorrect drop instillation technique, which could lead to higher rates of postoperative complications [3, 4]. Our study population, drawn from an urban academic centre, included a high proportion of racial minorities that have not been represented in previous studies of dropless cataract surgery. Across all patient populations, a dropless approach could improve patient satisfaction and outcomes by eliminating complex postoperative drop regimens [31–33]. For example, the slightly better visual improvement in the dropless group (logMAR 0.340 vs. 0.241, *p* < 0.01; approximately equivalent to 3 vs. 2 lines of improvement in Snellen) could be an indicator that consistent anti-inflammatory prophylaxis and avoidance of drop-related eye irritation marginally benefits visual recovery [3, 34]. Additionally, our diverse cohort suggests the dropless strategy is generalisable across different demographic groups not represented in previous studies.

Our study had several limitations. The retrospective design means there was no randomised assignment to treatments or strict standardisation of treatment protocol, allowing potential for selection bias. For example, surgeons might have chosen the dropless approach for certain patients and not others based on subjective criteria. Additionally, the dropless protocol was performed primarily by two surgeons, which may also bias outcomes. The disparity in treatment group sizes is another limitation, reflecting how the dropless protocol was a relatively new and selectively used protocol at our institution. The smaller dropless group sample size reduces power for some comparisons and prevented us from performing multivariable analyses or propensity score matching to control for confounding variables. Future studies with larger, more balanced cohorts may benefit from matching and multivariable modelling to strengthen statistical validity. Nonetheless, our study provides more diverse evidence to empower surgeons to select the best postoperative treatment regimens for their patients to help improve surgical outcomes and health equity. Future research should include prospective, randomised trials to control for confounding variables and determine ideal patient profiles for a dropless approach to cataract surgery.

## Summary

### What was known before

- Topical eye drops are the standard approach for controlling inflammation and infection after cataract surgery.
- Intraocular medications injected at the time of surgery, including steroids and antibiotics, have emerged as a “dropless” method that may improve medication adherence and simplify postoperative care.
- There is limited data comparing real-world outcomes of dropless and topical approached to cataract surgery, particularly in diverse patient populations.

### What this study adds

- This real-world retrospective cohort study found no significant differences in rates of intraocular pressure spikes, cystoid macular oedema, or rebound iritis between dropless and topical steroid regimens.
- The study cohort was pulled from a large, urban academic centre and included a higher proportion of Black patients, suggesting potential for broader accessibility and applicability of dropless cataract surgery.
- These findings support the noninferiority of sub-Tenon’s triamcinolone acetonide for inflammation prophylaxis following cataract surgery and underscore the need for further research to define optimal patient selection for a dropless approach.

## Data Availability

The datasets generated during the current study are not publicly available due protected health information but may be available from the corresponding author on reasonable request with proper authorisation from the institution.
